# PD-1 inhibitors plus chemotherapy for refractory EBV-positive DLBCL: a retrospective analysis

**DOI:** 10.1007/s44313-024-00042-6

**Published:** 2024-10-30

**Authors:** Youli Li, Yonghe Wu, Sufen Cao, Baohua Yu, Qunling Zhang, Zuguang Xia, Junning Cao, Fangfang Lv, Guang-Liang Chen

**Affiliations:** 1 Department of Medical Oncology, Fudan University Shanghai Cancer Center, Shanghai, 200032 P.R. China; 2 Department of Oncology, Shanghai Medical College, Fudan University, Shanghai, 200032 P.R. China; 3 Department of Medical Oncology, Fudan University Shanghai Cancer Center Xiamen Hospital, Xiamen, 361026 P.R. China; 4 Department of Pathology, Fujian Province, Zhangzhou Affiliated Hospital of Fujian Medical University, Zhangzhou, 363000 P.R. China; 5 Department of Pathology, Fudan University Shanghai Cancer Center, Shanghai, 200032 P.R. China; 6 Present Address: Department of Nursing, Fudan University Shanghai Cancer Center, Shanghai, 200032 P.R. China

**Keywords:** Epstein–Barr virus, Diffuse large B-cell lymphoma, Chemotherapy, PD-1 inhibitors, Treatment resistance, Immunotherapy

## Abstract

**Background:**

Immunochemotherapy has demonstrated a promising efficacy for a variety of B-cell lymphoma but has limited efficacy for Epstein–Barr virus-positive (EBV +) diffuse large B-cell lymphoma (DLBCL) that is refractory or relapsed to conventional chemotherapy regimens. Considering higher programmed death-ligand 1 (PD-L1) expression in the subset of patients with DLBCL with positive EBV, we speculated that PD-1 inhibitors plus chemotherapy may be an alternative regimen in patients with refractory/relapsed EBV + DLBCL.

**Methods:**

This retrospective study included six adult patients diagnosed with refractory EBV + DLBCL resistant to first-line immunochemotherapy regimens (R-CHOP). These patients received PD-1 inhibitors plus chemotherapy as second-line treatment.

**Results:**

The final analysis included six patients (four men and two women (median age, 50 years; range, 39–83 years)). Four patients were diagnosed with Epstein–Barr virus (EBV) + DLBCL, and two had DLBCL associated with chronic inflammation. Over a median follow-up of 20 months (range, 2–31 months), the objective response rate was 83% (5/6) and the complete remission rate was 67% (4/6). No severe immune-related adverse reactions occurred, and only a mild rash was reported, which did not necessitate the discontinuation of therapy.

**Conclusion:**

The combination of PD-1 inhibitors and chemotherapy offers promising results as a second-line treatment for patients with refractory EBV + DLBCL that is resistant to first-line immunochemotherapy regimens. These preliminary findings warrant further investigation in larger clinical trials to validate the efficacy and safety of this therapeutic approach.

## Introduction

Epstein–Barr virus-positive (EBV +) diffuse large B-cell lymphoma (DLBCL), which accounts for approximately 4–15% of cases, represents a particularly aggressive subset of B-cell lymphoma that predominantly affects older adults [1, 2]. This subtype is characterized by a poor prognosis, largely due to its resistance to conventional chemotherapeutic regimens [3, 4]. Effective therapeutic strategies for relapsed or refractory (R/R) EBV + DLBCL remain elusive, especially for patients who are not candidates for autologous stem cell transplantation. The rarity of EBV + DLBCL cases, coupled with regional disparities, presents significant challenges in conducting large-scale, high-quality clinical trials. These issues highlight the urgent need for innovative therapeutic approaches to manage this challenging lymphoma subtype [4].

Recent studies have highlighted the promise of combination immunotherapies, especially for patients with high programmed death-ligand 1 (PD-L1) expression, which is a common feature of EBV + DLBCL [3, 5–7]. Elevated PD-L1 levels in EBV + tumors have been linked to a more favorable response to PD-1 inhibitors [8, 9]. In vitro research and successful case reports have demonstrated that inhibiting the PD-1 pathway can restore T-cell functionality in EBV + DLBCL [8, 10]. The integration of chemotherapy with immune checkpoint inhibitors has been found to augment immune responses and enhances the effectiveness of the PD-1/PD-L1 blockade by facilitating antigen release through chemotherapy-induced cytotoxic cell death [11].

In light of these findings, we hypothesized that PD-1 inhibitors might show efficacy in refractory EBV + DLBCL. However, data on the efficacy of PD-1 inhibitors combined with chemotherapy as second-line therapy for R/R EBV + DLBCL remain limited.

## Patients and methods

### Patients and treatment

In this retrospective study, we evaluated six adult patients diagnosed with refractory EBV + DLBCL at our center who received a PD-1 inhibitor plus chemotherapy as rescue therapy between April 2019 and July 2021. The inclusion criteria were as follows: (1) pathologically diagnosed with DLBCL, (2) previously treated with R-CHOP or an R-CHOP-like regimen as first-line chemotherapy, (3) diagnosed with refractory DLBCL, (4) received at least two courses of PD-1 inhibitor plus chemotherapy, and (5) complete efficacy evaluation and follow-up information. The final follow-up evaluation was conducted in July 2023. The requirement for informed consent was waived for this retrospective study. All patient information was de-identified prior to data analysis. This study was approved by the Institutional Review Board of the Fudan University Shanghai Cancer Center (ZRB1612167-18).

Each treatment cycle consisted of a PD-1 inhibitor administered at a dose of 200 mg on the first day of each chemotherapy cycle consisting of 21 days. Chemotherapy regimens varied according to the individual condition of the patients; routine practice at our center is six–eight cycles, followed by maintenance using PD-1 inhibitor alone in patients who achieved complete response (CR), or partial response until disease progression, or death. Patients in the study did not undergo autologous hematopoietic stem cell transplantation due to old age and infirmity or refusal.

Primary refractory disease was defined as progression or an inconspicuous effect during first-line treatment, CR after first-line treatment, or progressive disease within 6 months of first-line treatment. Treatment efficacy was evaluated every two to four cycles during PD-1 inhibitor therapy in combination with chemotherapy and every 2 to 3 months during PD-1 inhibitor monotherapy, using 18F-deoxyglucose positron emission tomography-computed tomography (FDG-PET/CT)) or computed tomography (CT), in accordance with the 2014 Lugano classification [12]. Owing to the retrospective design, the timing of these scans varied, with most patients undergoing early evaluation after two–four cycles. Circulating EBV DNA was quantified by real-time quantitative polymerase chain reaction (qPCR). Overall survival (OS) was defined as the duration from the initiation of PD-1 inhibitor treatment to death or the last follow-up. Progression-free survival (PFS) was defined as the time from PD-1 inhibitor treatment to disease progression, death, or last follow-up, whichever occurred first.

### Immunohistochemical analysis

In situ hybridization (ISH) was used to examine the EBV status. The Hans algorithm was employed to classify the cell of origin as germinal center B-cell (GCB) or non-GCB [9].

## Results

### Clinical characteristics

The clinical and biological characteristics at diagnosis of the six patients, including four men and two women (median age, 50 years; range 39–83, years), are summarized in Table 1. Clinical stages were II (*n* = 2), III (*n* = 1), and IV (*n* = 3). Five patients had elevated lactate dehydrogenase levels. Four patients had an International Prognostic Index score > 2. Bulky disease was present in two patients.

**Table 1:** Baseline characteristics, first-line immunochemotherapy regimens and response of patients with refractory Epstein–Barr virus (EBV)-positive diffuse large B-cell lymphoma

Patient no	1	2	3	4	5	6
Age, years	46	60	83	48	51	39
Sex	Male	Female	Male	Male	Male	Female
Stage	II	III	IV	IV	IV	II
ECOG PS	1	1	1	3	2	1
Elevated LDH	No	Yes	Yes	Yes	Yes	Yes
IPI score	0	3	4	4	3	1
Bulky disease	Yes	No	No	No	No	Yes
Cell of origin	NA	Non-GCB	Non-GCB	Non-GCB	Non-GCB	Non-GCB
Immunochemothe-rapy(cycles)	R-CHOP (6)	R-CHOP (8)	R-miniCDOP (3)	R-miniCHOP (3)	R-CHOP (6)	R-CHOP(3), CHOP(2)
Response	PR	PD	PD	PD	PD	PR

*ECOG PS* Eastern Cooperative Oncology Group Performance Status, *LDH* lactate dehydrogenase, *IPI* International Prognostic Index, *GCB* germinal center B cell, *NA* not applicable, *R-CHOP* rituximab, cyclophosphamide, vindesine, doxorubicin, prednisone, *PR* partial response, *PD* progressive disease

### Histopathological and immunohistochemical analysis

Four patients were diagnosed with EBV + DLBCL, while two had DLBCL associated with chronic inflammation; all tested positive for EBV via ISH. Patients 2–6 were diagnosed with non-GCB-type DLBCL, while Patient 1 lacked data. Additionally, Patient 2 exhibited an 8q24/c-MYC translocation.

### Response to combination therapy with PD-1 inhibitors

**Fig. 1:**
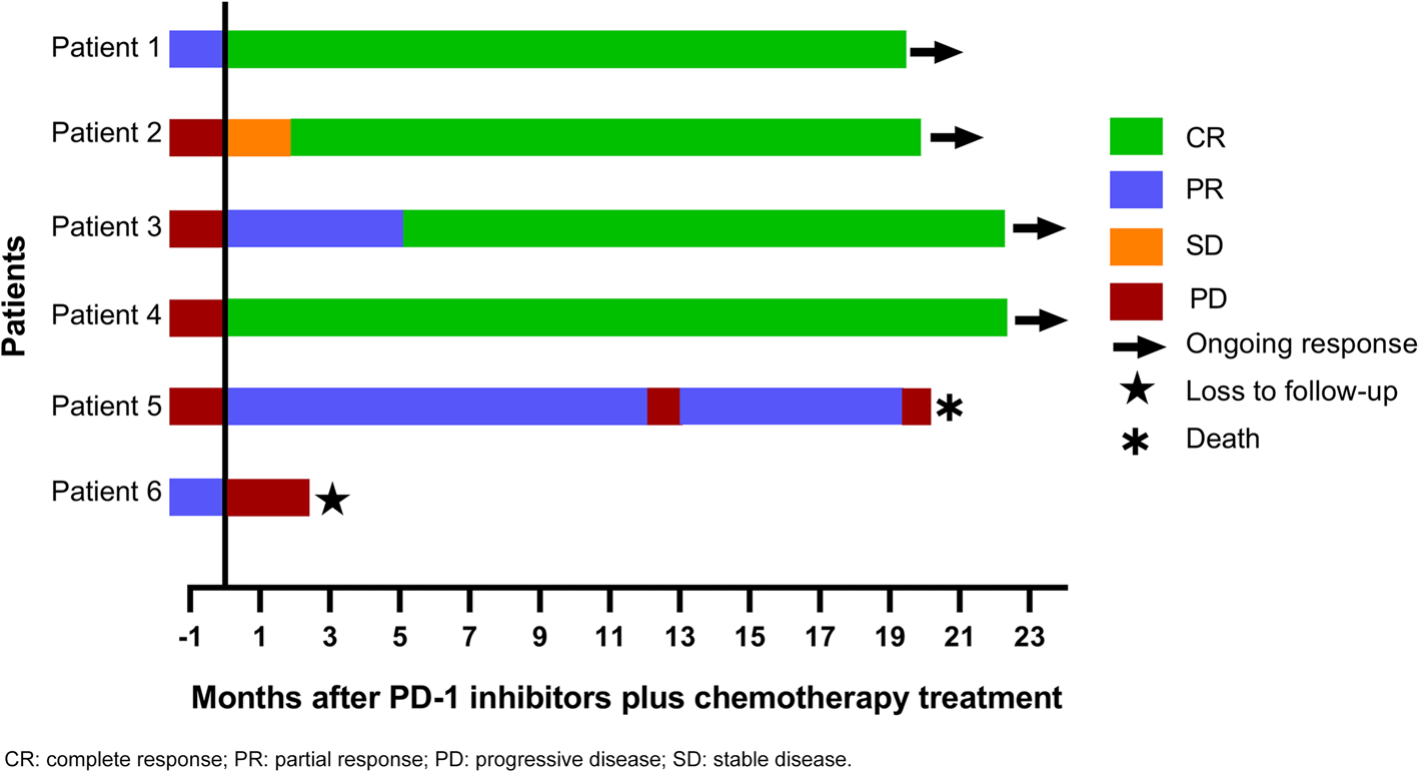
Programmed death-ligand 1 (PD-1) inhibitors plus chemotherapy treatment response and outcomes Five of the six patients achieved objective responses including four complete remissions and one partial remission following PD-1 blockade therapy. Patient 5 achieved partial response with a progression-free survival (PFS) of 10 months but subsequently experienced disease progression twice and ultimately died with an overall survival of 20 months. Patient 6 progressed after three cycles of immunotherapy with a PFS of 2 months and was subsequently lost to follow-up﻿.  CR: complete response; PR: partial response; PD: progressive disease; SD: stable disease

**Fig. 2:**
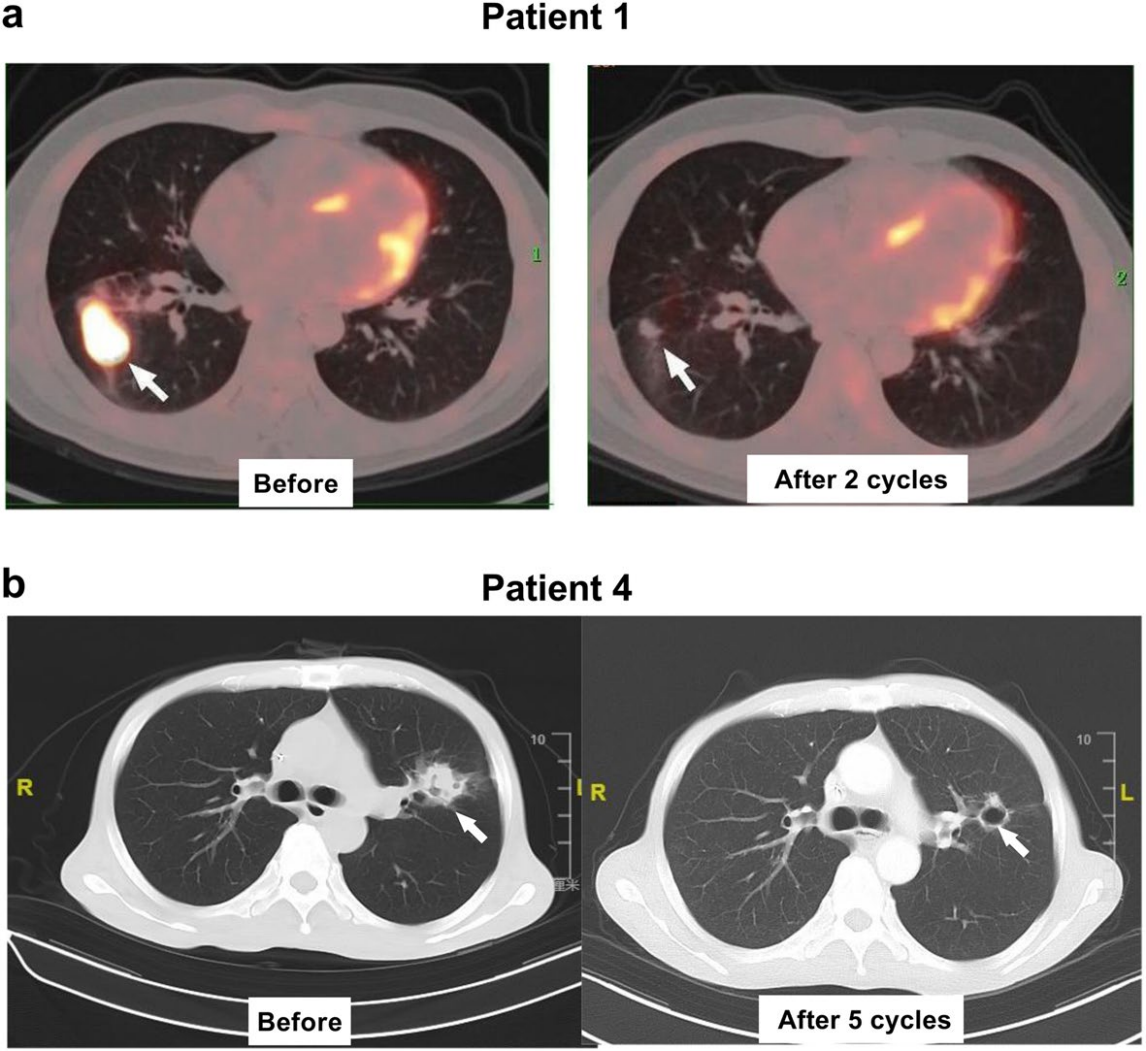
Pre- and post- programmed death-ligand 1 (PD-1) inhibitors plus chemotherapy treatment response on positron emission tomography (PET)/computed tomography (CT) or CT scans (**a**) Patient 1: Significant reduction in right lung lesion (white arrow) and complete response on PET/CT after two cycles (**b**) Patient 4: Most lesions (white arrows) undetectable after five cycles

**Fig. 3:**
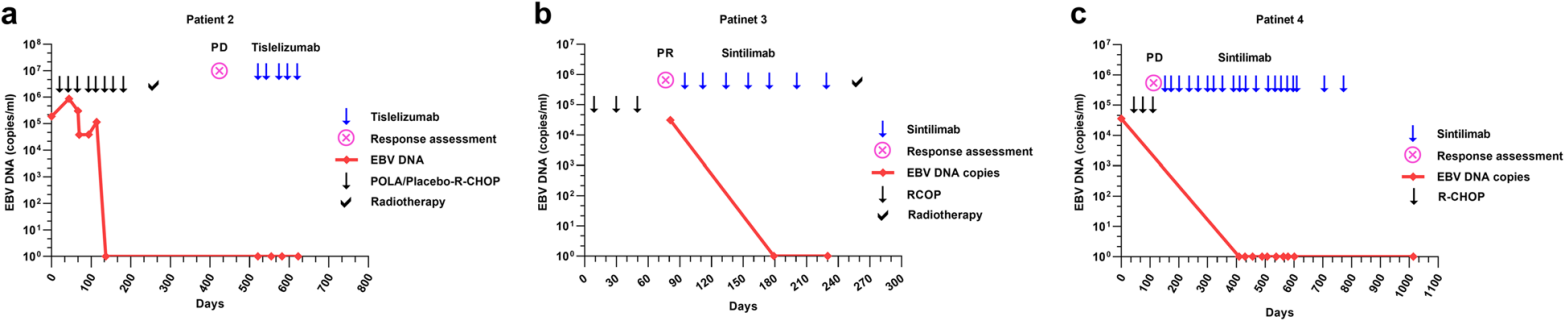
Changes in circulating Epstein–Barr virus (EBV) DNA levels with programmed death-ligand 1 (PD-1) inhibitors plus chemotherapy treatment (**a**) Patient 2: Ongoing remission with undetectable EBV DNA during treatment (**b**) Patient 3: Received sintilimab combined with gemcitabine and achieved partial response and then complete response (CR) with radiotherapy. EBV DNA decreased to undetectable levels (initial: 3.08x10^4^ copies/mL). **c** Patient 4: Achieved CR after five cycles, and the EBV DNA was monitored after 11 cycles of PD-1 inhibitor treatment and remained undetectable subsequently (initial: 3.56x10^4^ copies/mL)

Five of the six patients achieved objective responses after PD-1 inhibitor therapy. Four patients (Patients 1–4) achieved complete remission (CR)  with progression-free survival (PFS) rates of 19, 20, 22, and 31 months, respectively. One patient (Patient 5) achieved partial remission after three cycles of PD-1 inhibitor therapy with a PFS of 10 months, but experienced disease progression twice and ultimately died with an OS of 20 months. One patient (Patient 6) progressed after three cycles of immunotherapy with a PFS of 2 months and was subsequently lost to follow-up (Fig. 1). Details of the treatment responses are provided in Table 2. The pre- and post- PD-1 inhibitors plus chemotherapy treatment responses on positron emission tomography PET/CT or CT scans of Patients 1 and 4 are shown in Fig. 2. The EBV DNA levels of Patient 2 remained undetectable during subsequent PD-1 inhibitor therapy (Fig. 3a). Circulating EBV DNA levels in Patients 3 and 4 dropped from high copy number values to undetectable values after PD-1 inhibitor treatment (Fig. 3b, c). Dynamic EBV DNA monitoring was not performed in other patients.

**Table 2:** The outcomes of PD-1 inhibitors plus chemotherapy in patients with refractory EBV-positive diffuse large B-cell lymphoma

Patient no	1	2	3	4	5	6
PD-1 inhibitors (cycles)	Tislelizumab (32)	Tislelizumab (5)	Sintilimab (8)	Sintilimab (16)	Sintilimab (18)	Camrelizumab (3)
Combined chemotherapy regimens	R-CHOP × 2	R-GP × 5	G × 6	R-ICE × 6	P × 8; G × 6	R-CHOP × 3
Response	CR	CR	CR	CR	PR	PD
Final response	CR	CR	CR	CR	PD	PD
PFS (months)	19 +	20 +	22 +	31 +	10	2
OS (months)	19 +	20 +	22 +	31 +	20	2 +
irADRs (grade)	None	None	None	None	Skin rash (1)	None

*R-CHOP* rituximab, cyclophosphamide, vindesine, doxorubicin, prednisone, *R-GP* rituximab, gemcitabine, cisplatinum, *R-ICE* rituximab, ifosfamide, carboplatin, and etoposide, *G* gemcitabine, *P* cisplatinum, *CR* complete response, *PR* partial response, *PD* progressive disease, *OS* Overall Survival, *PFS* progression-free survival, *irADRs* immune-related adverse drug reactions

### Immune-related adverse drug reactions

Only Patient 5 experienced a Grade 1 rash, which did not impede further combination therapy with PD-1 inhibitors. No immune-related adverse drug reactions were observed in the other patients.

## Discussion

EBV + DLBCL is an aggressive malignancy which is associated with immune evasion [3, 8, 13]. This subtype shows significant resistance to conventional chemotherapeutic strategies and is linked to poorer OS rates [3, 14, 15]. Patients with refractory EBV + DLBCL exhibit poor responses to first-line treatment, and the efficacy of second-line regimens for R/R DLBCL is currently unsatisfactory. The application of immune checkpoint inhibitors as monotherapies for R/R DLBCLs has been largely unsatisfactory in recent years [16, 17]. However, evidence suggests that combining PD-1/PD-L1 inhibitors with other therapeutic modalities offers promising results [6, 7, 18, 19]. This study explored the efficacy of PD-1 inhibitors combined with chemotherapy as a second-line salvage therapy for refractory EBV+ DLBCL. 

Our findings revealed that this combination yielded favorable outcomes, achieving an objective response rate (ORR) of 83% (5/6) and a complete response rate (CRR) of 67% (4/6). In a study by Smith et al.,combining Pembrolizumab with the R-CHOP regimen in patients with untreated DLBCL demonstrated strong efficacy, with an ORR of 90%, a CRR of 77%, and a 2-year PFS rate of 83% [6]. This study aligns with our findings that PD-1 inhibitors combined with chemotherapy represent an effective treatment for DLBCL. Additionally, a retrospective study found that PD-1 inhibitors combined with the ICE (ifosfamide, carboplatin, and etoposide) regimen provided a well-tolerated and effective salvage therapy for patients with R/R DLBCL, achieving an ORR of 62.7% and a CRR of 43.3% [7]. Moreover, of the three patients with EBV infection in this study, two achieved PR, one achieved CR, and the PFS was 5.5, 26.4, and 30.1 months, respectively. Patients with positive EBV seemed to be benefit more from the P-ICE regimen. PD-1 inhibitors combined with chemotherapy demonstrated efficacy in R/R DLBCL, particularly in those who are EBV + , which is in line with our findings. Our study suggests that targeting the PD-1/PD-L1 pathway with inhibitors may be an effective strategy for this challenging subset of DLBCL, especially given the generally well-tolerated nature of the treatment regimen, with no serious adverse events reported.

EBV + DLBCL in patients aged > 50 years is associated with shorter OS [20] and is often linked to the non-GCB subtype [3, 13]. This subtype, coupled with increased PD-L1 expression and CD30 positivity, generally indicates poorer survival outcomes [13, 21–23]. In our cohort, all patients characterized with the non-GCB subtype responded poorly to the first-line R-CHOP regimen. Furthermore, despite initial predictions of favorable prognoses for young (< 50 years old) patients with EBV + DLBCL [20], Patients 1 and 4 exhibited poor responses to first-line immunochemotherapy. Nevertheless, they achieved complete and sustained responses following administration of PD-1 inhibitors and chemotherapy. Notably, among our six cases, two patients (Patients 3 and 5) with positive EBV were diagnosed with DLBCL associated with chronic inflammation [24]. This condition is particularly distinct in its pathogenesis and clinical features compared with other malignant lymphomas [25]. These two patients responded to PD-1 inhibitor treatment plus chemotherapy. Therefore, employing PD-1 monoclonal antibodies as a second-line treatment for some young patients with refractory EBV + DLBCL and DLBCL associated with chronic inflammation may represent a promising therapeutic approach.

The efficacy of brentuximab vedotin (BV), an anti-CD30 monoclonal antibody, has been significant in treating R/R EBV + and CD30-positive non-Hodgkin lymphomas, with an ORR of 48% and a median response duration of 10.1 months [26]. The results of the ECHELON-3 study showed that BV in combination with lenalidomide and rituximab in patients with R/R DLBCL significantly improved the OS, PFS, and ORR [27]. Given the correlation between high CD30 expression and poor outcomes in patients with EBV + DLBCL [23], BV represents a promising option for this subset of patients. Prior research supports the combination of PD-1 inhibitors with anti-CD30 antibodies in classical Hodgkin's lymphoma [28, 29], suggesting similar potential benefits for EBV + DLBCL. Applying this combined approach to R/R EBV + DLBCL presents encouraging prospects. Additionally, the ongoing exploration of innovative therapies, such as bispecific antibodies, CAR-T cell treatments, and small-molecule inhibitors that target specific pathways and biological processes, remains crucial [30].

This study has some limitations that warrant caution when interpreting the results. Firstly, a single-center retrospective study with a limited sample size is the main limitation of this study, which may affect the generalizability of the findings. A larger cohort is necessary to confirm our results and enhance the robustness of the conclusions. Secondly, the retrospective design resulted in missing baseline data and irregular monitoring of prognostic markers, which may potentially affect data reliability. Although EBV + DLBCL is characterized by increased PD-L1 expression, our retrospective approach meant that PD-L1 expression levels were not assessed in some patients, and further measurements were absent when the disease became refractory. EBV DNA serves as a biomarker reflecting the disease status of EBV + DLBCL and correlates with prognosis. However, there were no uniform monitoring and follow-up criteria due to the retrospective nature of the study, which hindered the ability to adequately represent the changing trends in EBV DNA in each patient. Thus, a future prospective study is essential for a thorough evaluation. Lastly, although all patients initially received R-CHOP or R-CHOP-like chemotherapy, subsequent treatment regimens varied, incorporating PD-1 inhibitors and various chemotherapy protocols. This variability reflects the lack of standardized treatment and follow-up protocols, which may be attributed to diverse patient populations, including differences in age and physical status. In the future, uniform inclusion and exclusion criteria, along with standardized treatment protocols, will need to be established. However, we observed a positive response to PD-1 inhibitors combined with chemotherapy in refractory EBV + DLBCL, a condition for which data on effective therapies are scarce, making our findings valuable for clinical practice.

## Conclusion

In summary, this retrospective study demonstrates that PD-1 inhibitors combined with chemotherapy are a well-tolerated and effective second-line rescue therapy for refractory EBV + DLBCL. However, further exploration of combination therapies with larger cohorts and additional prospective, multicenter clinical research is necessary to validate these findings.

## Data Availability

This article’s underlying data cannot be shared publicly for ethical/privacy reasons.

## Abbreviations

EBV +: Epstein-Barr virus-positive
DLBCL: Diffuse large B-cell lymphoma
R/R: Relapsed or refractory
PD-L1: Programmed death-ligand 1
OS: Overall survival
IHC: Immunohistochemistry
ISH: In situ hybridization
GCB: Germinal center B-cell
EBERs: EBV-encoded small RNAs
FDG- PET/CT: 18F-deoxyglucose positron emission tomography-computed tomography
CT: Computed tomography
qPCR: Quantitative polymerase chain reaction
PFS: Progression-free survival
CR: Complete remission
PR: Partial remission
R-CHOP: Rituximab, cyclophosphamide, doxorubicin, vincristine, prednisone
R-ICE: Rituximab, ifosfamide, carboplatin, etoposide
ICE: Ifosfamide, carboplatin, etoposide
PD: Progression of disease
ORR: Objective response rate
CRR: Complete response rate
BV: Brentuximab vedotin
ECOG PS: Eastern Cooperative Oncology Group Performance Status
LDH: Lactate dehydrogenase
IPI: International Prognostic Index
irADRs: Immune-related adverse drug reactions
